# Upregulation of RSPO3 via targeted promoter DNA demethylation inhibits the progression of cholangiocarcinoma

**DOI:** 10.1186/s13148-023-01592-9

**Published:** 2023-11-07

**Authors:** Guanhua Wu, Da Wang, Fei Xiong, Wenzheng Liu, Qi Wang, Junsheng Chen, Bing Wang, Yongjun Chen

**Affiliations:** Department of Biliary-Pancreatic Surgery, Tongji Hospital, Tongji Medical College, Huazhong University of Science and Technology, 1095 Jiefang Road, Wuhan, 430074 Hubei China

**Keywords:** RSPO3, DNA methylation, TET1, Targeted demethylation, Precise treatment

## Abstract

**Background:**

Cholangiocarcinoma (CCA) refers to a collection of malignant tumors that develop from the biliary epithelium. Extensive clinical evidence and epidemiological observations indicate a concerning increase in both the incidence and mortality rates of CCA. Surgical resection is currently the sole available cure for CCA. However, it is unfortunate that only a fraction of patients has access to surgery at the time of diagnosis. Moreover, there is a high incidence of cancer recurrence after resection, and systemic treatments have limited efficacy. Therefore, the identification of novel biomarkers for CCA-targeted molecular therapy remains a crucial task in oncology research.

**Results:**

Our study demonstrated that low expression of RSPO3 was associated with poorer survival rates in patients with CCA. We found that the RSPO3 promoter DNA was hypermethylated in CCA, which was correlated with the low expression of RSPO3. The expression of RSPO3 was influenced by the balance between the DNA methyltransferase DNMT3a and the DNA demethylase TET1 in CCA. In vitro and in vivo experiments showed that targeting RSPO3 promoter DNA methylation using dCas9DNMT3a promoted tumorigenicity of CCA, while targeted RSPO3 promoter DNA demethylation using dCas9TET1CD inhibited CCA tumorigenicity. Additionally, in our primary CCA model, knockdown of Rspo3 promoted CCA progression, whereas overexpression of Rspo3 inhibited CCA progression.

**Conclusions:**

Our findings suggest that increased methylation and decreased expression of RSPO3 may indicate a poor prognosis in CCA. Restoring RSPO3 expression by targeting promoter DNA demethylation could offer insights for precise treatment of CCA.

**Supplementary Information:**

The online version contains supplementary material available at 10.1186/s13148-023-01592-9.

## Introduction

Cholangiocarcinoma (CCA) refers to a collection of malignant tumors that develop from the biliary epithelium. This type of cancer is known for its high aggressiveness, as evidenced by a 95% fatality rate within 5 years and a contribution of approximately 2% to overall cancer-related deaths [1–3]. There is a substantial amount of clinical evidence and epidemiological observations that indicate a rising trend in the morbidity and mortality rates of CCA [2, 4, 5]. Given the limited presence of symptoms during the initial stages of the disease, CCA is typically diagnosed at a more advanced stage [6]. Currently, surgical resection remains the sole curative option; however, it is regrettable that this procedure is accessible to less than 30% of patients. Furthermore, there is a considerable risk of cancer recurrence, and systemic treatments offer limited benefits [7, 8]. Therefore, identifying novel biomarkers for targeted molecular therapy in CCA is a crucial endeavor in the field of oncology research.

R-spondin3 (RSPO3) encodes a secretory signaling protein with the same name. It is involved in several signaling pathways, including the Wnt/β-catenin pathway [9], the AMPKα signaling pathway [10], and the Erk signaling pathway [11]. Growing evidence suggests that RSPO3 can play a role in tumorigenesis and development [12]. RSPO3 has been identified as a driver of various cancers, such as bladder cancer, breast cancer, and ovarian cancer [13–15]. However, several studies have indicated that RSPO3 functions as a tumor progression suppressor [12, 16]. Increased RSPO3 expression has been linked to a favorable prognosis in various cancer types, and the introduction of RSPO3 into tumors has been found to mitigate tumor progression [17, 18]. Nevertheless, the precise biological role of RSPO3 in CCA remains uncertain.

According to reports, a significant correlation has been observed between the expression of RSPO3 and the DNA methylation status of its own promoter region [19]. In general, DNA methylation is primarily catalyzed by DNA methyltransferases (DNMTs). In humans, three typical isoforms of DNMTs have been identified: DNMT1, DNMT3a, and DNMT3b. DNMT1 plays a crucial role in maintaining methylation, while DNMT3a and DNMT3b are involved in de novo methylation [20]. DNMT3a and DNMT3b are enzymes that play a crucial role in carcinogenesis by actively adding methyl groups to new DNA sequences, thereby controlling gene expression [21]. The process of DNA demethylation is classified into two categories: 'passive' and 'active' demethylation, as per the model proposed [22]. Passive demethylation refers to the inability of DNMT1 to fully methylate the 5-C site on the daughter strand during semiconserved DNA replication in cell division. This leads to a gradual decrease in genome-wide methylation, hence the term 'passive'. On the other hand, active DNA demethylation involves the action of the dioxygenase 10–11 translocation (TET) family, which includes three members: TET1, TET2, and TET3 [23].

Our study revealed a significant association between low expression of RSPO3 and poorer survival in patients with CCA. We found that the RSPO3 promoter DNA was hypermethylated in CCA, which correlated with the low expression of RSPO3. The expression of RSPO3 was determined by the balance between the DNA methyltransferase DNMT3a and the DNA demethylase TET1 in CCA. In this study, we conducted in vitro and in vivo experiments to investigate the effects of targeted RSPO3 promoter DNA methylation and demethylation on the tumorigenicity of CCA. Our findings revealed that targeted RSPO3 promoter DNA methylation using dCas9DNMT3a enhanced CCA tumorigenicity, while targeted RSPO3 promoter DNA demethylation using dCas9TET1CD inhibited CCA tumorigenicity. Additionally, in our primary CCA model, we observed that knockdown of Rspo3 promoted CCA progression, whereas overexpression of Rspo3 inhibited CCA progression.

## Materials and methods

### Patient samples

Human CCA tissue samples were obtained from the Department of Biliary and Pancreatic Surgery at Tongji Hospital, Huazhong University of Science and Technology in Wuhan, China. These samples were diagnosed as CCA through pathological examination following surgical resection. Patient follow-up was conducted after surgery, and the date of death or last follow-up was recorded. The procedures for collecting patient tissue samples were ethically approved by the Party Committee Technology of Tongji Hospital, Huazhong University of Science and Technology, and adhered to the principles of the Declaration of Helsinki.

### Cell culture

Human CCA cells (TFK1, QBC939, EGI1, HuCCT1, HuH28, RBE, SSP25, HCCC9810) were cultured in RPMI 1640 medium (HyClone, USA) supplemented with 10% fetal bovine serum (NEWZERUM, New Zealand), 100 units/ml penicillin, and 100 μg/ml streptomycin. The cells were incubated in a cell culture incubator at 37 °C with 5% CO_2_. QBC939 and HCCC9810 cells were treated with 5 μM or 10 μM decitabine (DAC) (#S1200, Selleck Chemicals, Shanghai, China) for 72 h or 96 h with daily change of complete medium.

### Plasmid construction

The full-length sequences of DNMT1, DNMT3a, DNMT3b, DNMT3l, TET1, TET2, and TET3 were amplified by PCR from human cell cDNAs and cloned into pHAGE-Flag vector. Gene-specific sequence small hairpin RNA primers, provided by Sangon Biotech, were inserted into the pLKO.1 vector. The target methylation or target demethylation plasmids, sgRNA-dCas9DNMT3a and sgRNA-dCas9TET1CD, were constructed by recombination of PCR-amplified DNMT3a sequence and TET1CD sequence into PX458-dCas9. The sgRNA targeting RSPO3 was inserted into dCas9DNMT3a and sgRNA-dCas9TET1CD after BbsI enzymatic digestion of the plasmid. The empty vector sgRNA-dCas9DNMT3a was used as a control for targeted methylated control experiments, while the empty vector sgRNA-dCas9TET1CD was used as a control for demethylated experiments. The primers used are shown in Additional file 1.

### Viral packaging and viral infection

The target gene plasmid was co-transfected with pMD2G and psPAX packaging plasmids in HEK293T cells. The cells were cultured in DMEM high glucose medium (HyClone, USA) supplemented with 10% fetal bovine serum (NEWZERUM, New Zealand). After 72 h, the supernatants were collected using a 0.45 μm filter (BS-PES-45, Biosharp, China). TFK1 and QBC939 cells were then treated with 1 ml of viral supernatant and 1 ml of RPMI 1640 complete medium, along with 2 μl of polybrene (40804ES76, Yeasen, China), for 24 h. Subsequently, the cells were screened with puromycin hydrochloride (CL13900, Selleck, USA) at a concentration of 1 μg/ml for 2 weeks. Finally, the reverse transcription quantitative polymerase chain reaction (RT-qPCR) was conducted to assess the efficiency of target gene knockdown or expression.

### Cell proliferation assay

Cell viability was assessed using the Cell Counting Kit (CCK-8) (40203ES92, Yeasen, China). A total of 1000 cells were plated into 96-well plates and incubated at 37 °C with 5% CO_2_. CCK-8 reagent was then added to the wells following the manufacturer's instructions. Absorbance at 450 nm was measured using the Thermo Scientific™ Multiskan™ FC instrument (Thermo Fisher, Shanghai, China). For colony formation assays, 1000 cells were grown in 6-well plates with 2 ml of fresh medium and incubated for 2 weeks. Visible colonies were stained with 0.5% crystal violet, and the number of colonies was counted.

### Total RNA isolation and reverse transcription real-time PCR

Total RNA was extracted using the RNA isolator Total RNA extraction reagent (TRIzol, Vazyme, Nanjing, China). cDNA was extracted using HiScript®III RT SuperMix for RT-qPCR (Vazyme) following the manufacturer's protocol. ChamQ Universal SYBR RT-qPCR Master Mix RT-qPCR (Vazyme) was utilized, with β-actin serving as the internal reference. The analysis of the results was performed using Bio-Rad CFX Manager 2.1. Additional file 1 provides information about the primers used.

### Bisulfite sequencing PCR (BSP)

BSP primers were designed using the online MethPrimer program (http://www.urogene.org/methprimer). Primers information is found in Additional file 1. DNA extraction was performed using a DNA extraction kit (D3396020000J12T006, Omega, USA), and the BSP transformation reaction was carried out using a kit (EM101-02, Vazyme, China). PCR amplification was then conducted using Taq enzyme (C601-02, Vazyme, China). The PCR products were purified for vector ligation (EM101-02, Vazyme, China), and a total of 10 bacterial clones were randomly selected for sequencing.

### Immunohistochemical (IHC) staining

Tumor tissue was embedded in paraffin and sectioned to prevent desiccation. The sections were then baked at 70 °C for 2 h. Deparaffinization with xylene and hydration with gradient ethanol were carried out. Antigen repair was performed by treating the sections with sodium citrate solution in boiling water for 20 min. To prevent nonspecific binding, a 5% BSA solution was applied for 30 min. The sections were then incubated with the primary antibody at 4 °C for 12 h. Nuclei were visualized using diaminobenzidine for 1 min and stained with hematoxylin. Differentiation was achieved by treating the sections with hydrochloric acid in ethanol, followed by dehydration with gradient ethanol, rinsing with xylene, and air drying. The sections were sealed with neutral adhesive and observed under a light microscope. Images were captured for analysis. The number of cancer cells expressing the target protein was counted, and correlation analysis was performed.

### Experiments with animals

BALB/c (nu/nu) male nude mice and male C57BL/6J mice were obtained from Jiangsu GemPharmatech and housed in a specific pathogen-free facility at the Animal Center of Tongji Hospital, Tongji Medical College, Huazhong University of Science and Technology. For xenograft experiments, 5-week-old male BALB/c (nu/nu) nude mice were randomly assigned to groups (*n* = 8 per group). 3 × 10^6^ cells were suspended in 100 μl PBS and injected subcutaneously into the dorsal side of the mice. Tumor measurements were recorded weekly, and the tumor volume was calculated using the formula: total tumor volume (mm^3^) = 0.52 × length × width × width. After 5 weeks, the mice were euthanized by dislocating the cervical vertebrae, and the tumors were surgically removed for further analysis.

A primary CCA mouse model was created by diluting 20 μg of pT3-EF1aH-myr-Akt and 20 μg of pT3-EF1aH-NICD1 together with 6 μg of transposon plasmid pCMV(CAT)T7-SB100 in 2.0 mL of saline. The resulting plasmid mixture (2 mL) was injected into the lateral tail vein of 5-week-old male C57BL/6J mice within 7 s. To achieve RSPO3 knockdown, the plasmid pT3-EF1aH was converted into pT3-U6, and a small hairpin RNA specific for the Rspo3 gene was designed and ligated into the pT3-U6 vector. An additional 20 μg of pT3-U6-Rspo3#1, pT3-U6-Rspo3#2 plasmid, or the corresponding control plasmid pT3-U6 was added to the above plasmid mixture. To overexpress Rspo3, the pT3-EF1aH-Rspo3 plasmid was constructed using pT3-EF1aH. The pT3-EF1aH-Rspo3 plasmid or the corresponding control plasmid pT3-EF1aH was added to the above plasmid mixture and injected into the lateral caudal vein of male C57BL/6J wild-type mice. Livers were collected 4 or 5 weeks after hydrodynamic transfection for analysis of CCA tumorigenesis and progression.

### Statistical analysis

All experiments were conducted independently, with each experiment repeated at least three times. The data are presented as the mean ± standard deviation, unless otherwise specified. Statistical analysis was performed using GraphPad Prism software 8.0 (GraphPad Prism Software Inc., San Diego, CA, USA). The Student's t-test was used to compare two independent groups. Survival curves were generated using the Kaplan–Meier method and analyzed using the log-rank test. Statistical significance was defined as *p* values < 0.05. The levels of significance were indicated as follows: *p < 0.05, **p < 0.01, ***p < 0.001.

## Results

### RSPO3 promoter DNA methylation and expression status in CCA

**Fig. 1:**
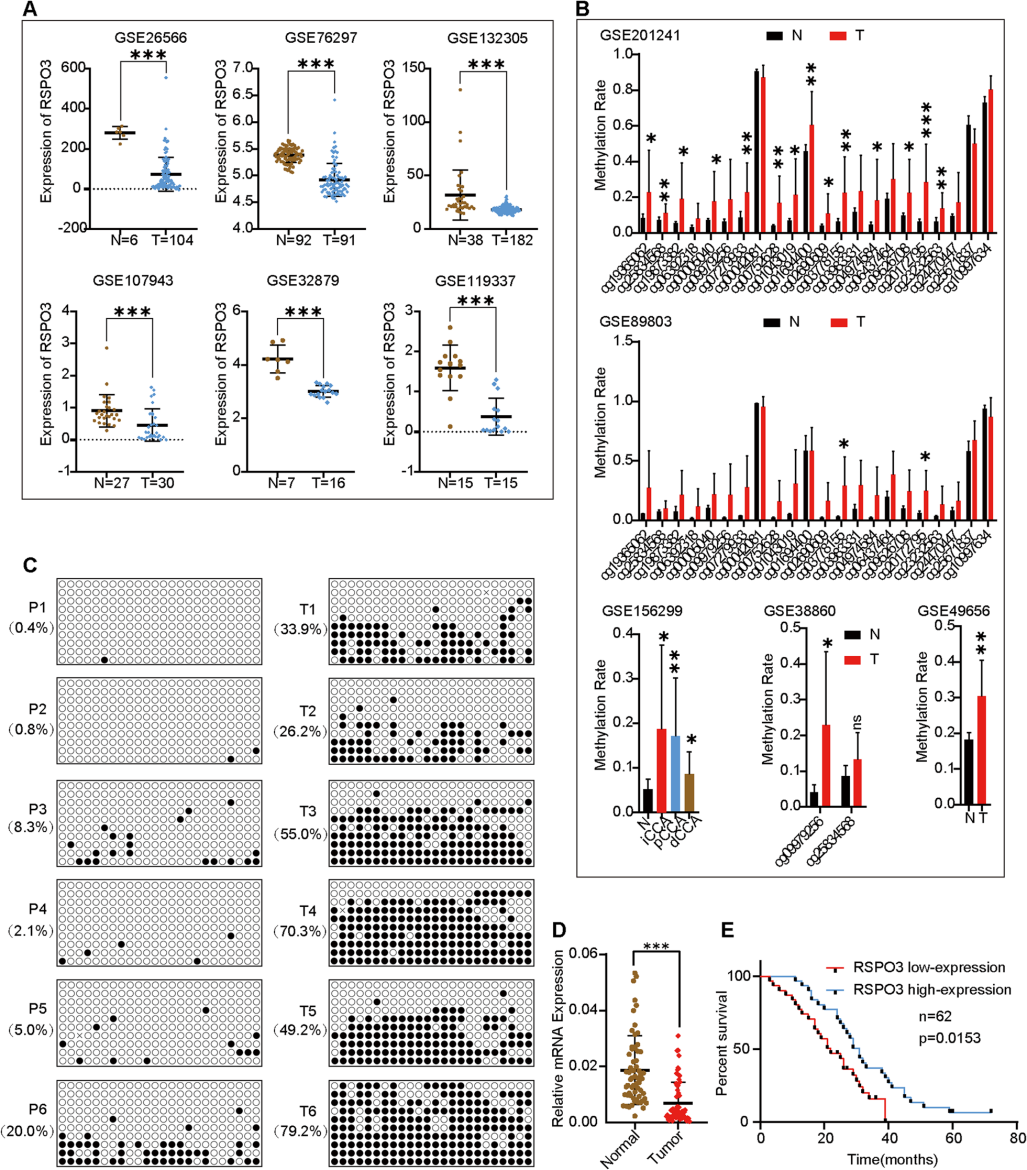
RSPO3 promoter DNA methylation and expression status in CCA. **A** The expression level of RSPO3 in CCA (represented by T in the figure) and adjacent tissue (including normal bile duct or non-tumor tissue, represented by N in the figure) was analyzed using datasets GSE26566, GSE76297, GSE132305, GSE107943, GSE32879, and GSE119337. **B** The DNA methylation difference of RSPO3 in CCA and adjacent tissues (including normal bile duct or non-tumor tissue) was analyzed in multiple datasets GSE201241, GSE89803, GSE156299, GSE38860, and GSE49656. In the figure, CCA is represented by T and adjacent tissues are represented by N. Methylation levels in the range of 0–1, with larger values representing higher methylation rates. **C** DNA methylation of the RSPO3 promoter in 6 pairs of in CCA and adjacent tissues. **D** RT-qPCR analysis of RSPO3 expression in 62 patients with CCA and adjacent tissues. **E** Kaplan–Meier curves showing overall survival of CCA patients stratified by median RSPO3 expression (high and low expressing tumors; *n* = 62)

In this study, we investigated the characterization of RSPO3 in cholangiocarcinoma (CCA) by analyzing the GEO database. Our analysis revealed that RSPO3 expression was significantly downregulated in CCA compared to adjacent tissues (Fig. 1A). Additionally, we found that RSPO3 gene promoter methylation was significantly higher in human CCA tissues than adjacent tissues (Fig. 1B). To further validate these findings, we examined six pairs of CCA and adjacent tissues and observed that RSPO3 promoter methylation in CCA was significantly higher than in adjacent tissues (Fig. 1C). Furthermore, we performed RT-qPCR on 62 pairs of CCA tissue and adjacent tissues obtained from our hospital. The results indicated that the expression of RSPO3 was downregulated in CCA tissues compared to adjacent tissues (Fig. 1D), suggesting that DNA methylation inhibits RSPO3 transcription in human CCA. Subsequently, we classified the expression of RSPO3 as either 'high' or 'low' based on the median value. Notably, our findings demonstrated that low RSPO3 mRNA expression was associated with a worse prognosis compared to high expression (Fig. 1E).

### RSPO3 expression is regulated by DNA methylation in CCA

**Fig. 2:**
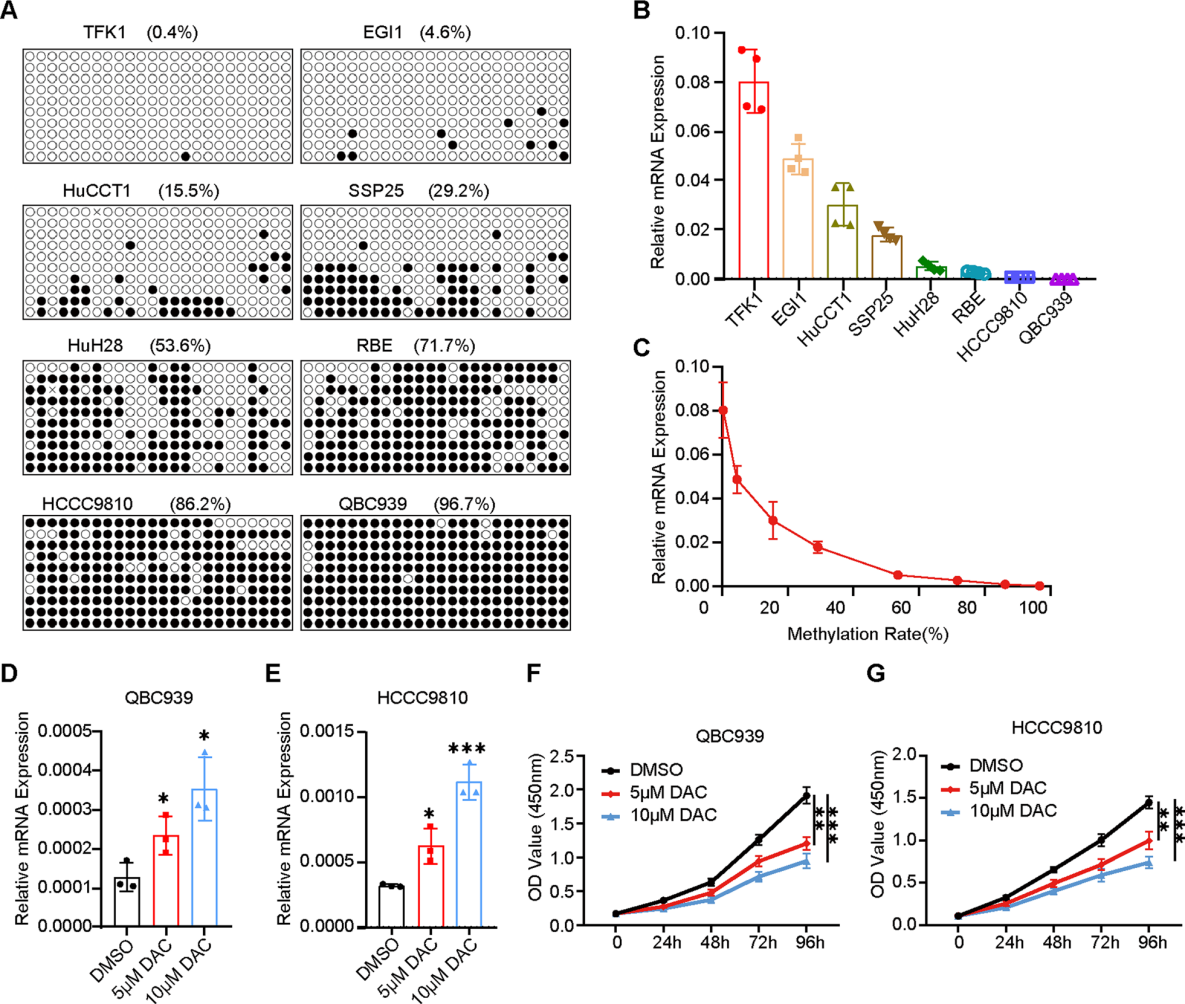
RSPO3 expression is regulated by DNA methylation in CCA. **A** Differential DNA methylation of RSPO3 in CCA cell lines. **B** The expression level of RSPO3 in CCA cell lines. **C** Expression levels of RSPO3 in CCA cell lines as related to its promoter methylation. **D**, **E** Expression of RSPO3 mRNA in CCA cells QBC939 and HCCC9810 after DAC treatment. Data are shown normalized to β-actin. **F**, **G** The proliferation of DAC-treated CCA cells QBC939 and HCCC9810 was performed using Cell Counting Kit-8 (CCK-8)

To investigate the relationship between RSPO3 promoter methylation and its mRNA expression, we examined RSPO3 expression and its promoter methylation by bisulfite sequencing PCR (BSP) of genomic DNA from CCA cells (TFK1, EGI1, QBC939, HuCCT1, HuH28, RBE, HCCC9810, and SSP25) after bisulfite processing (Fig. 2A). The relative expression levels of RSPO3 were also examined in these cells (Fig. 2B). Our findings revealed that RSPO3 mRNA expression levels decreased as the methylation of its promoter DNA increased (Fig. 2C). Decitabine (DAC) is a cytosine analog that functions as a broad-spectrum DNA methyltransferase inhibitor. It covalently traps DNA methyltransferases, which place the important epigenetic mark 5-methyl-2'-deoxycytidine by methylating 2'-deoxycytidine (dC) at the C5 position. This leads to DNA hypomethylation [24]. To further validate this relationship, we conducted concentration-dependent treatment with DAC dosing in CCA cells QBC939 and HCCC9810, which have higher methylation levels. We observed a significant elevation in the mRNA expression of RSPO3 (Fig. 2D, E). Additionally, the proliferative capacity of QBC939 and HCCC9810 decreased in a concentration-dependent manner after DAC dosing treatment, as determined by CCK-8 experiments (Fig. 2F, G).

### DNMT3a-mediated RSPO3 DNA hypermethylation promotes CCA growth

**Fig. 3:**
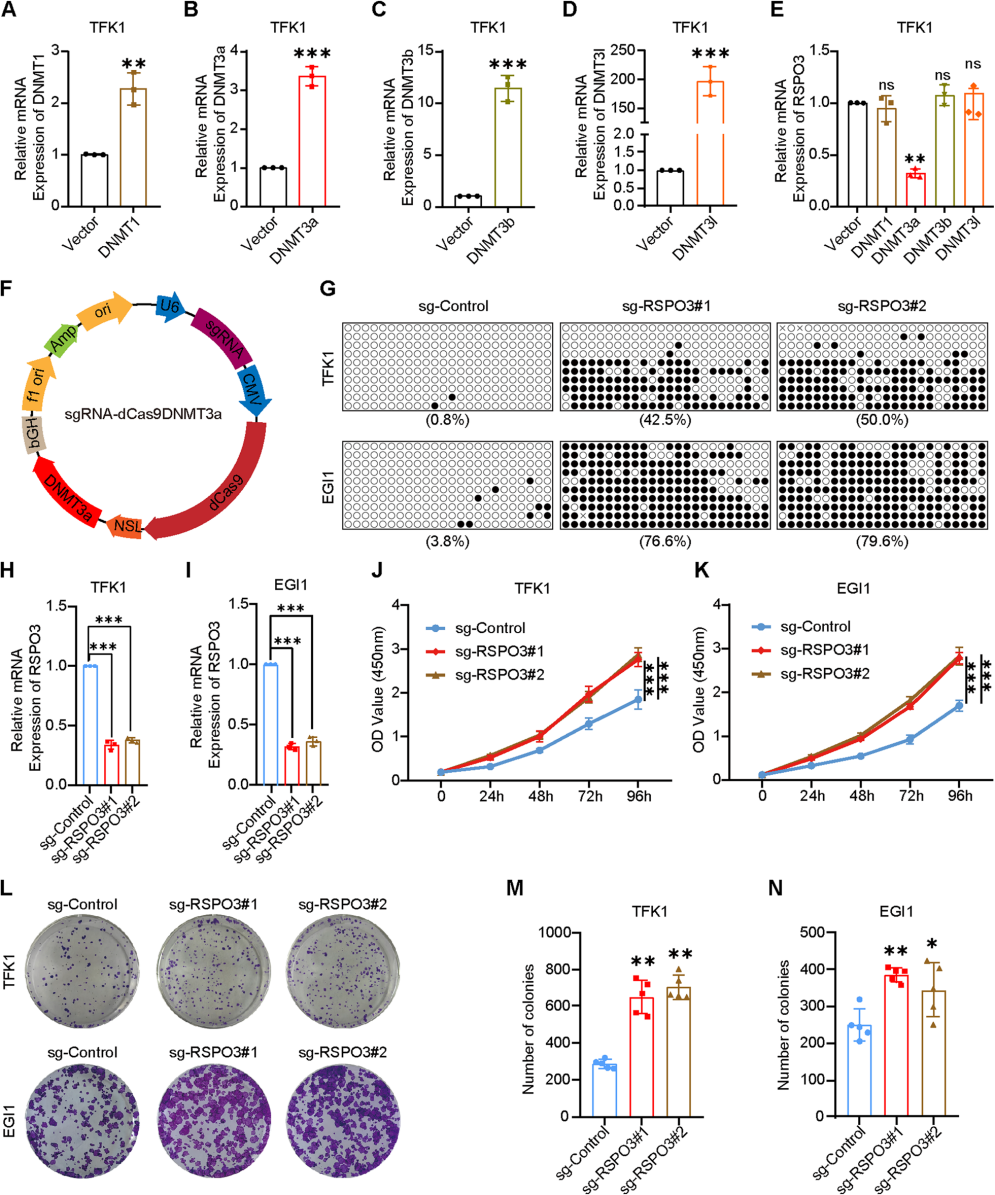
DNMT3a-mediated RSPO3 DNA hypermethylation promotes CCA growth. **A**–**D** Overexpression of DNMT1, DNMT3a, DNMT3b and DNMT3l in TFK1 cells confirmed by RT-qPCR. Data are shown after normalization to β-actin. **E** Expression of RSPO3 in DNMT1, DNMT3a, DNMT3b and DNMT3l overexpression TFK1 cells. Data are shown after normalization to β-actin. **F** An illustration of targeted methylation by sgRNA-dCas9DNMT3a. **G** Differential DNA methylation targeting RSPO3 promoter methylation in CCA cell lines TFK1 and EGI1. **H**,** I** RSPO3 expression after targeted RSPO3 promoter methylation in CCA cell lines TFK1 and EGI1. **J**,** K** Cell proliferation after methylation of the targeted RSPO3 promoter in the CCA cell lines TFK1 and EGI1 was performed using Cell Counting Kit-8 (CCK-8) assay. **L**–**N** Cell proliferation after methylation of the targeted RSPO3 promoter in the CCA cell lines TFK1 and EGI1 was performed using clone formation assay

To investigate the DNA methyltransferase priming catalysis that results in promoter DNA methylation in RSPO3, we constructed DNA methyltransferase overexpression vectors for DNMT1, DNMT3a, DNMT3b, and DNMT3l. These overexpression of DNA methyltransferases in TFK1 cells was confirmed using RT-qPCR. (Fig. 3A–D). Specifically, the overexpression of DNMT3a led to a reduction in the mRNA expression level of RSPO3 (Fig. 3E). Therefore, we hypothesized that DNMT3a acts as the DNA methyltransferase of RSPO3 in CCA. To investigate this, we constructed the vector sgRNA-dCas9DNMT3a targeting DNA methylation (Fig. 3F) and constructed two target sequences sg-RSPO3#1 and sg-RSPO3#2 that target the RSPO3 promoter. In CCA cells TFK1 and EGI1, targeting by sg-RSPO3#1 and sg-RSPO3#2 led to a noteworthy rise in DNA methylation of the RSPO3 promoter (Fig. 3G). The RT-qPCR results demonstrated a significant decrease in the mRNA levels of sg-RSPO3#1 and sg-RSPO3#2 following the targeting of RSPO3 promoter DNA methylation (Fig. 3H, I). The proliferative capacity of TFK1 and EGI1 cells, which were targeted with RSPO3 promoter DNA methylation in CCA cells, was observed to be significantly increased according to the results of the CCK-8 assay (Fig. 3J, K) and clone formation assay (Fig. 3L–N). To ensure the exclusion of off-targets outside the target gene, we utilized the online tool Benchling (https://www.benchling.com/) to identify the top 6 potential off-target loci (Additional file 2: Fig. S1A, F). We then tested the mRNA expression levels of these off-target genes to determine if their transcript levels were influenced by off-target effects. The results indicated that sg1 and sg2 of sgRNA-dCas9DNMT3a were effective and specific for RSPO3 methylation (Additional file 2: Figure S1B, C, G, H).

### TET1-mediated demethylation of RSPO3 inhibits CCA growth

**Fig. 4:**
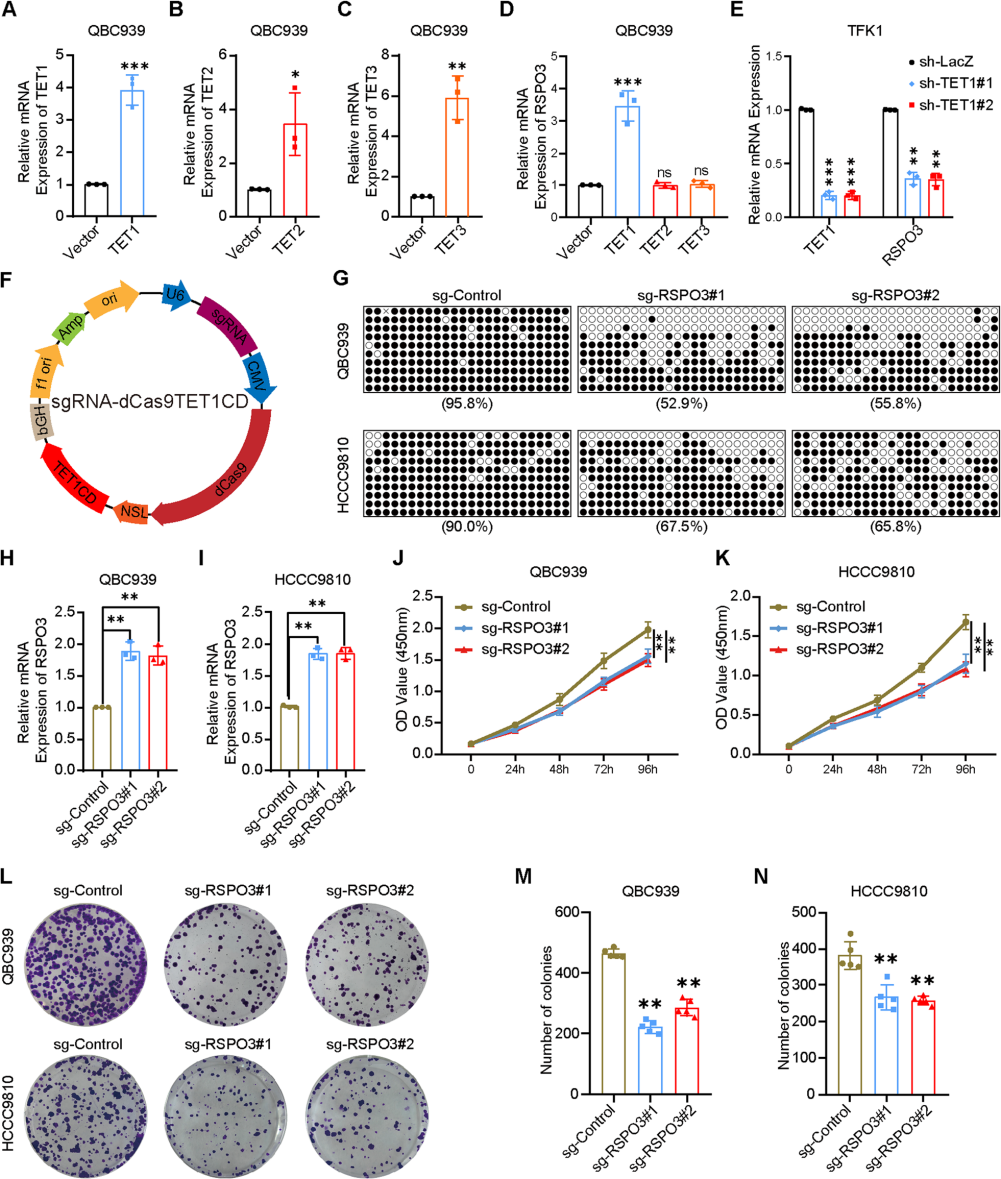
TET1-mediated demethylation of RSPO3 inhibits CCA growth. **A**–**C** Overexpression of TET1, TET2 and TET3 in QBC939 cells confirmed by RT-qPCR. Data are shown after normalization to β-actin. **D** Expression of RSPO3 in TET1, TET2 and TET3 overexpression QBC939 cells. Data are shown after normalization to β-actin. **E** Knockdown of TET1 in TFK1 cells resulted in lowered expression of RSPO3 confirmed by RT-qPCR. Data are shown after normalization to β-actin. **F** An illustration of targeted methylation by sgRNA-dCas9TET1CD. **G** Differential DNA methylation targeting RSPO3 promoter methylation in CCA cell lines QBC939 and HCCC9810. **H**,** I** RSPO3 expression after targeted RSPO3 promoter methylation in CCA cell lines QBC939 and HCCC9810. **J**,** K** Cell proliferation after methylation of the targeted RSPO3 promoter in the CCA cell lines QBC939 and HCCC9810 was performed using Cell Counting Kit-8 (CCK-8) assay. **L**–**N** Cell proliferation after methylation of the targeted RSPO3 promoter in the CCA cell lines QBC939 and HCCC9810 was performed using clone formation assay

To investigate the DNA demethylase responsible for promoter hypomethylation of RSPO3, we constructed overexpression vectors for TET1, TET2, and TET3. The overexpression of these DNA demethylase vectors in QBC939 cells, which have high DNA methylation of the RSPO3 promoter, was confirmed through RT-qPCR (Fig. 4A–C). The results indicated that only TET1 overexpression led to an increase in the mRNA expression level of RSPO3 (Fig. 4D). To further validate our findings, we observed a decrease in RSPO3 mRNA expression levels following the knockdown of TET1 in TFK1 cells with low DNA methylation (Fig. 4E). In this study, we hypothesized that TET1 functions as the DNA demethylase of RSPO3 in CCA. To investigate this, we constructed a demethylation vector called sgRNA-dCas9TET1CD (Fig. 4F). Two target sequences, sg-RSPO3#1 and sg-RSPO3#2, were subsequently constructed to target the RSPO3 promoter. Targeting sg-RSPO3#1 and sg-RSPO3#2 led to a notable decrease in RSPO3 promoter DNA methylation in CCA cells QBC939 and HCCC9810 (Fig. 4G). The RT-qPCR results indicated a significant upregulation in the mRNA levels of sg-RSPO3#1 and sg-RSPO3#2 following the targeting of RSPO3 promoter DNA demethylation (Fig. 4H, I). The study observed that targeting RSPO3 promoter DNA demethylation in CCA cells QBC939 and HCCC9810 resulted in a significant decrease in their proliferative capacity, as demonstrated by the CCK-8 assay (Fig. 4J, K) and clone formation assay (Fig. 4L–N). Similarly, we analyzed the mRNA expression levels of six potential off-target loci and tested these off-target genes in the mRNA expression levels. Our findings revealed that sg1 and sg2 of sgRNA-dCas9TET1CD were effective and specific for RSPO3 demethylation (Additional file 2: Fig. S1D, E, I, J)

### RSPO3 as a CCA suppressor by targeting methylation/demethylation of RSPO3 in vivo

**Fig. 5:**
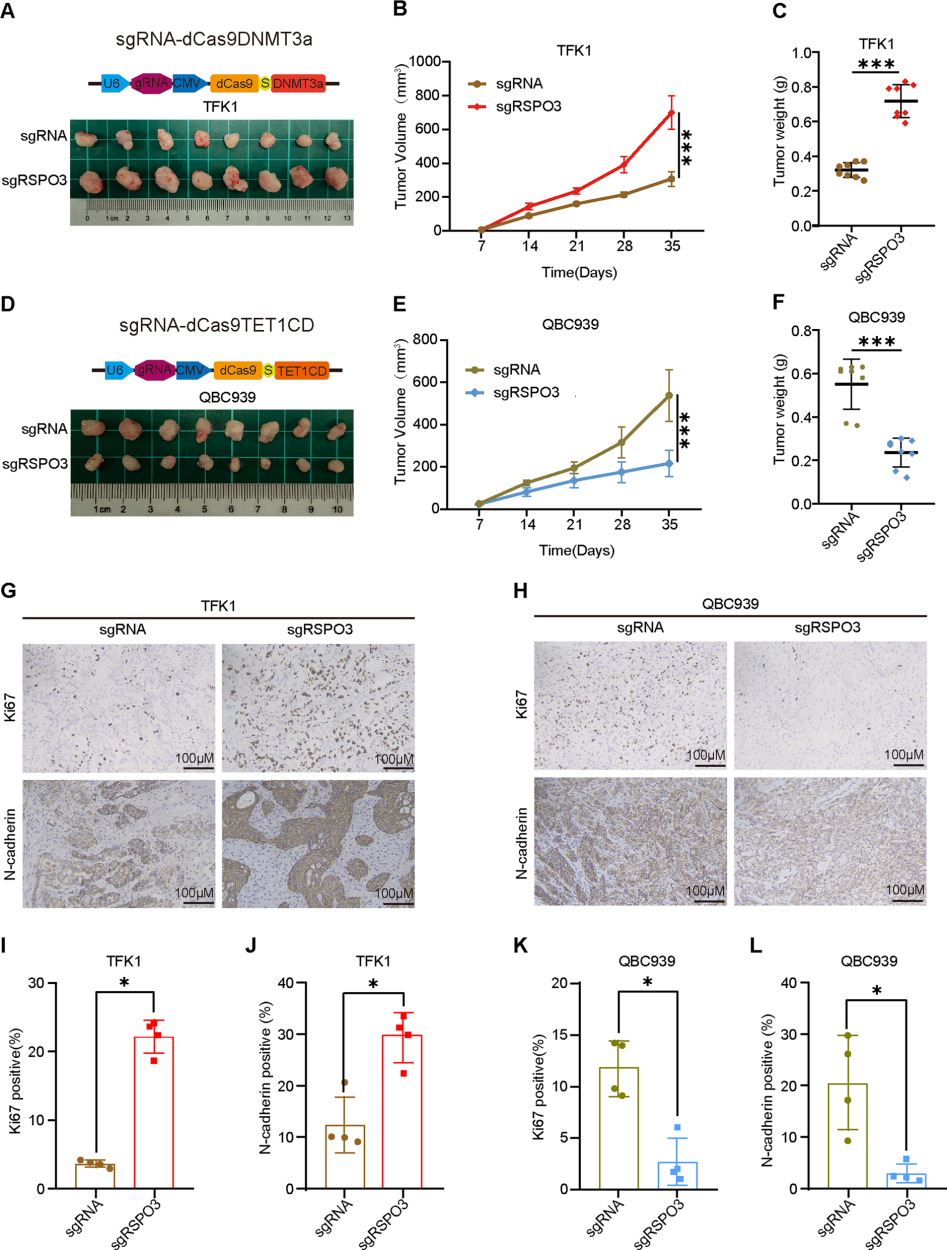
RSPO3 as a CCA suppressor by targeting methylation/demethylation of RSPO3 in vivo.** A** Overview of tumors in xenografts of RSPO3 targeted methylated and control cells in vivo (*n* = 8). **B**, **C** Tumor volume and end-stage tumor weight after injection of TFK1 cells transfected with sgRNA-dCas9DNMT3a and sg-RSPO3-dCas9DNMT3a. **D** Overview of tumors in xenografts of RSPO3 targeted demethylated and control cells in vivo (*n* = 8). **E**,** F** Tumor volume and end-stage tumor weight after injection of QBC939 cells transfected with sgRNA-dCas9TET1CD and sg-RSPO3-dCas9TET1CD. **G**–**L**. Immunohistochemical staining was performed to detect proliferation and infiltration of tumor tissue. Percentages of Ki67 and N-cadherin positive cells were analyzed

To investigate the effects of targeting promoter DNA methylation and demethylation of RSPO3 on CCA growth in vivo, we conducted experiments using TFK1 cells transfected with sg-RSPO3#1-dCas9DNMT3a and QBC939 cells transfected with sg-RSPO3#1-dCas9TET1CD, along with their respective control cells. These cells were injected into female wild-type BALB/c nude mice to establish xenografting models. Our results demonstrated that targeted methylation of RSPO3 significantly increased TFK1 tumor volume (Fig. 5A), which also resulted in accelerated tumor growth and increased weight (Fig. 5B, C). In this regard, targeted demethylation of RSPO3 significantly reduced QBC939 tumor volume (Fig. 5D), which was also evident in slower tumor growth and lower tumor weight (Fig. 5E, F). Targeted demethylation of RSPO3 significantly inhibited the growth of CCA cells. Ki67 immunohistochemical staining of tumors targeting RSPO3-methylated TFK1 revealed an increased number of proliferating cells (Fig. 5G, I). Fewer proliferating cells were observed in tumors targeting RSPO3 demethylated QBC939 (Fig. 5H, K). N-cadherin reflects the infiltration ability of tumor cells, and immunohistochemical staining by N-cadherin showed that the degree of tumor infiltration increased in tumors targeting RSPO3-methylated TFK1 (Fig. 5G, J). In contrast, tumors targeting RSPO3 demethylated QBC939 had a lower degree of tumor infiltration (Fig. 5H, L). Overall, the data suggest that targeting demethylated RSPO3 as a therapeutic strategy has potential antitumor effects in CCA.

### Rspo3 as a tumor suppressor in primary CCA model

**Fig. 6:**
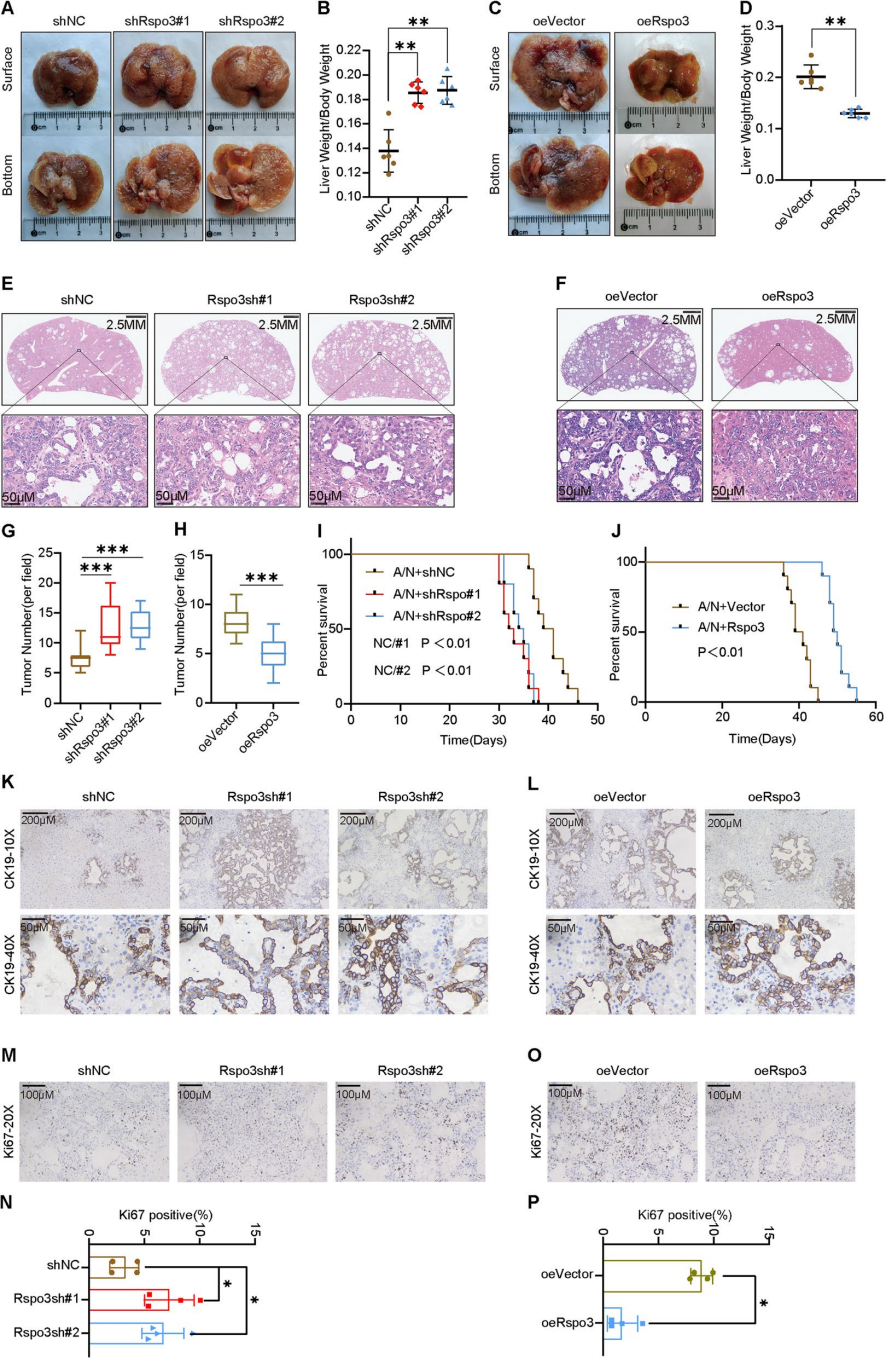
Rspo3 as a tumor suppressor in primary CCA model.** A** Representative overview of liver tumor load in shNC, shRspo3#1 and shRspo3#2 mice. **B** Ratios of liver weight to body weight in shNC, shRspo3#1, and shRspo3#2 mice groups. **C** Representative overview of liver tumor load in oeVector and oeRspo3 mice. **D** Ratios of liver weight to body weight in oeVector and oeRspo3 mice groups. **E**,** G** H&E analysis of the number of primary CCAs in shNC, shRspo3#1, and shRspo3#2 mice groups. **F**,** H** H&E analysis of the number of primary CCAs in oeVector and oeRspo3 mice groups. **I** Kaplan–Meier curves showing overall survival in the shNC, shRspo3#1, and shRspo3#2 primary CCA model mice groups.** J** Kaplan–Meier curves showing overall survival in the oeVector and oeRspo3 primary CCA model mice groups. **K**,** L** Immunohistochemical staining of cytokeratin 19 in primary CCA model. **M**–**P**. Immunohistochemical staining was performed to detect proliferation of primary CCA tissue. Percentages of Ki67 positive cells were analyzed

Aiming to further confirm the role of Rspo3 in regulating CCA progression in vivo, we established a mouse model of CCA primary tumors by hydrodynamic transfection of activated forms of Akt and NICD1 plasmids into C57BL/6 mice. The Rspo3 knockdown plasmid was co-injected with the Akt/NICD1 plasmid to investigate whether knockdown of Rspo3 had a role in promoting the progression of CCA in vivo. Notably, Akt/NICD1/ Rspo3 knockdown (shRspo3#1, shRspo3#2) mice showed more lesions in mouse livers 4 weeks after plasmid injection compared to controls (shNC) (Fig. 6A). The ratio of liver weight to body weight in mice showed a significant increase in Rspo3 knockdown tumor load (Fig. 6B). The Rspo3 overexpression plasmid was co-injected with the Akt/NICD plasmid to see whether Rspo3 had an inhibiting effect on the progression of CCA in vivo. Remarkably only a few small lesions were seen in the livers of Akt/NICD1/ Rspo3 mice 5 weeks after plasmid injection compared with controls (Fig. 6C). The ratio of liver weight to body weight in mice showed a significant decreased tumor load with Rspo3 overexpression (Fig. 6D). Hematoxylin and eosin (H&E) staining showed a significant increase in the number of tumor lesions in Rspo3 knockdown mice compared to control mice (Fig. 6E, G). In contrast, the number of tumor lesions was significantly reduced in Rspo3 overexpressing mice compared to control compared mice (Fig. 6F, H). Survival outcomes were consistently poorer in Rspo3 knockdown mice (Fig. 6I) and consistently superior in Rspo3 overexpressing mice (Fig. 6J) compared with their respective controls. Cytokeratin 19 (CK19) in liver is a marker to differentiate between CCA and hepatocellular carcinoma, which stains negatively in hepatocytes and positively staining due to CCA originating from epithelial. Immunohistochemical showed positive staining for CK19 in all CCA tumor cells induced by transfection with Akt/NICD1 (Fig. 6K, L). More proliferating cells were observed in the tumors of Rspo3 knockdown mice as revealed by Ki67 immunohistochemical staining (Fig. 6M, N), and fewer proliferating cells were observed in the primary CCA of Rspo3 overexpressing mice (Fig. 6O, P). Overall, our data demonstrate the function of Rspo3 in inhibiting CCA tumorigenesis and progression in vivo.

## Discussion

The R-spondin (RSPO) family consists of four genes, namely RSPO1, RSPO2, RSPO3, and RSPO4, which encode secretory signaling proteins with class-based names [16, 25]. The receptors LGR4/5/6 and RSPOs are found in various organs, indicating that RSPOs may play a role in regulating stem cells in multiple parts of the body. RSPO3 has been reported to be associated with muscle fiber development, fat distribution, and the progression of acute myeloid leukemia [26–28]. However, the expression level of RSPO3 in cholangiocarcinoma (CCA) and its regulatory effect on tumor proliferation remain unknown. In this study, we analyzed GEO data and clinical tissue information to discover that RSPO3 expression was downregulated in CCA. Furthermore, we observed that increased expression of RSPO3 inhibited the progression of CCA, as evidenced by cell proliferation assays conducted in nude mice xenograft models and primary CCA models.

Epigenetic alterations, such as DNA methylation, have a strong association with cancer progression [29]. Downregulation of tumor suppressor genes is often linked to transcriptional silencing mediated by DNA methylation [30]. Demethylation refers to the process of inhibiting the addition of methyl groups to cytosine DNA bases. This is particularly relevant in cases of genome-wide hypomethylation, where the excessive methylation of cytosine DNA bases can lead to the development of cancer. Demethylation has emerged as a potential therapeutic strategy for cancer treatment [31]. In this study, we observed that the expression of RSPO3 increased when the methylation rate of the CpG site in the RSPO3 promoter region decreased. Additionally, we found a negative correlation between RSPO3 promoter methylation and RSPO3 expression in CCA, confirming that the downregulation of RSPO3 expression was mediated by hypermethylation of the RSPO3 promoter. Furthermore, we observed that RSPO3 expression was upregulated in CCA cells following treatment with the broad-spectrum demethylating drug DAC. DNA methyltransferase inhibitors, such as 5-aza-2-deoxycytidine, have demonstrated significant inhibitory effects on DNMT3a, DNMT3b, and DNMT1. These inhibitors have been approved for the treatment of hematologic tumors and are now being explored for their potential in treating solid tumors [32]. However, its nonspecific effect on decreasing methylation throughout the genome can lead to genomic instability, thereby increasing the risk of tumor proliferation and infiltration [33]. As a result, targeted methylation or demethylation for precision therapy has emerged as a more effective approach for tumor treatment.

DNA methyltransferases (DNMTs) play a crucial role in the formation and maintenance of DNA methylation patterns. The DNMT family consists of DNMT1, DNMT3a, DNMT3b, and DNMT3l. During mammalian development, DNA methylation can be lost passively or actively demethylated. The active demethylation process involves the ten-eleven translocation (TET) dioxygenase family, which includes TET1, TET2, and TET3 [29]. DNMT3a is a de novo DNA methyltransferase that plays a crucial role in epigenetic programming in various types of tumors [34]. In this study, we provide evidence that DNMT3a plays a role in the regulation of RSPO3 methylation in CCA cells. Our findings demonstrate that targeting RSPO3 promoter DNA methylation using sg-RSPO3-dCas9DNMT3a leads to a decrease in RSPO3 expression and promotes CCA progression. Aberrant expression of methylcytosine dioxygenase TET1 is involved in epigenetic reprogramming and tumor progression [35]. This study demonstrates that TET1 plays a role in regulating RSPO3 demethylation in CCA cells. By targeting the RSPO3 promoter DNA demethylation with sg-RSPO3-dCas9TET1CD, the expression of RSPO3 is elevated and the progression of CCA is inhibited. These findings suggest that the balance between DNA methyltransferase DNMT3a and demethylase TET1 determines the level of DNA demethylation in the RSPO3 promoter, which in turn affects the progression of CCA. Additionally, the antitumor effects of sg-RSPO3-dCas9TET1CD in CCA were demonstrated both in vitro and in vivo.

In summary, DNA hypermethylation of the RSPO3 promoter region can lead to the downregulation of RSPO3 expression, thereby contributing to the development of CCA. Furthermore, we demonstrated that targeting the methylation level of the RSPO3 promoter region using sg-RSPO3-dCas9TET1CD resulted in the restoration of RSPO3 expression and exhibited significant antitumor effects in both in vitro and in vivo experiments. Overall, our investigation sheds light on the biological role of RSPO3 in CCA, offering a novel target for its development and presenting a new approach for early diagnosis and precise treatment of CCA.

### Supplementary Information

**Additional file 1**. Primer sequences used for plasmid construction, RT-qPCR and BSP assay.

**Additional file 2**. The Off-target detection of dCas9-based methylation/demethylation.

## Data Availability

All data generated or analyzed during this study are included in this published article and its Additional files 1 and 2.
