# Supplementary material for: Upregulation of RSPO3 via targeted promoter DNA demethylation inhibits the progression of cholangiocarcinoma
Source: Clin Epigenetics. 2023 Nov 7;15:177. doi: 10.1186/s13148-023-01592-9 (PMC10629118; doi:10.1186/s13148-023-01592-9)
Supplement: Supplementary file 1 — Additional file 1. Primer sequences used for plasmid construction, RT-qPCR and BSP assay. [file 13148_2023_1592_MOESM1_ESM.docx]

Table S1. Primers for plasmid construction

| Gene | Sequence |
| --- | --- |
| DNMT1-F | CGGATCGGGTTTAAACGGATCCGCCACCATGCCGGCGCGTACCGCCCCGA |
| DNMT1-R | GCCCTCTAGACTCGAGCGGCCGCTAGTCCTTAGCAGCTTCCTC |
| DNMT3a-F | CGGATCGGGTTTAAACGGATCCGCCACCATGCCCGCCATGCCCTCC |
| DNMT3a-R | GCCCTCTAGACTCGAGCGGCCGCACACACGCAAAATACTCCTTCAG |
| DNMT3b-F | CGGATCGGGTTTAAACGGATCCGCCACCATGAAGGGAGACACCAGGCATC |
| DNMT3b-R | GCCCTCTAGACTCGAGCGGCCGTTCACATGCAAAGTAGTCCTTCAG |
| DNMT3l-F | CGGATCGGGTTTAAACGGATCCGCCACCATGGCGGCCATCCCAGCCCTG |
| DNMT3l-R | GCCCTCTAGACTCGAGCGGCCGTAAAGAGGAAGTGAGTTCTGTTG |
| TET1-F | CGGATCGGGTTTAAACGGATCCGCCACCATGTCTCGATCCCGCCATGC |
| TET1-R | GCCCTCTAGACTCGAGCGGCCGGACCCAATGGTTATAGGGCCC |
| TET2-F | CGGATCGGGTTTAAACGGATCCGCCACCATGGAACAGGATAGAACCAACC |
| TET2-R | GCCCTCTAGACTCGAGCGGCCGTATATATCTGTTGTAAGGCCCTG |
| TET3-F | CGGATCGGGTTTAAACGGATCCGCCACCATGAGCCAGTTTCAGGTGCC |
| TET3-R | GCCCTCTAGACTCGAGCGGCCGGATCCAGCGGCTGTAGGG |
| shTET1#1F | CCGGCCTATATGTATGGCACAATATCTCGAGATATTGTGCCATACATATAGGTTTTTG |
| shTET1#1R | AATTCAAAAACCTATATGTATGGCACAATATCTCGAGATATTGTGCCATACATATAGG |
| shTET1#2F | CCGGACACAACTTGCTTCGATAATTCTCGAGAATTATCGAAGCAAGTTGTGTTTTTTG |
| shTET1#2R | AATTCAAAAAACACAACTTGCTTCGATAATTCTCGAGAATTATCGAAGCAAGTTGTGT |
| sgRSPO3#1F | CACCGTGGCTGGCATCGCCGCGAAC |
| sgRSPO3#1R | AAACGTTCGCGGCGATGCCAGCCAC |
| sgRSPO3#2F | CACCGGCTCCTGGAACCCCGGTTCG |
| sgRSPO3#2R | AAACCGAACCGGGGTTCCAGGAGCC |
| shRspo3#1F | CCGGCAGCGAGACAAGAACTTGTATCTCGAGATACAAGTTCTTGTCTCGCTGTTTTTG |
| shRspo3#1R | CATGCAAAAACAGCGAGACAAGAACTTGTATCTCGAGATACAAGTTCTTGTCTCGCTG |
| shRspo3#2F | CCGGCGAGACAAGAACTTGTATAGTCTCGAGACTATACAAGTTCTTGTCTCGTTTTTG |
| shRspo3#2R | CATGCAAAAACGAGACAAGAACTTGTATAGTCTCGAGACTATACAAGTTCTTGTCTCG |
| Rspo3-F | GTGTGGTGGAATTCTGCAGATAGCCACCATGCACTTGCGACTGATTTCTTG |
| Rspo3-R | GATCAGCGGGTTTAAACCATGGCTAGTGTACAGTGCTGACTGATAC |

Table S2. Primers for RT-qPCR

| Gene | Sequence |
| --- | --- |
| ACTB-F | CATGTACGTTGCTATCCAGGC |
| ACTB-R | CTCCTTAATGTCACGCACGAT |
| DNMT1-F | AGAACGGTGCTCATGCTTACA |
| DNMT1-R | CTCTACGGGCTTCACTTCTTG |
| DNMT3a-F | GTCATGTGGTTCGGAGACGG |
| DNMT3a-R | AGTGTCACTCTCATCGCTGTC |
| DNMT3b-F | AGGGAAGACTCGATCCTCGTC |
| DNMT3b-R | GTGTGTAGCTTAGCAGACTGG |
| DNMT3l-F | ATGAAGTCAAGGCTAACCAGC |
| DNMT3l-R | CGTCATCGTCGTACAGGAAGAG |
| TET1-F | CAGAACCTAAACCACCCGTG |
| TET1-R | TGCTTCGTAGCGCCATTGTAA |
| TET2-F | TCAGCATCATCAGCATCACA |
| TET2-R | ACTCACCCATCGCATACCTC |
| TET3-F | GCCGGTCAATGGTGCTAGAG |
| TET3-R | CGGTTGAAGGTTTCATAGAGCC |
| RSPO3-F | TGTGCAACATGCTCAGATTACA |
| RSPO3-R | TGCTTCATGCCAATTCTTTCCA |
| KDELC2-F | TGGAGCATCGAAAAGTCAATGG |
| KDELC2-R | CATACGTTGGAAGGACAACATCT |
| FADD-F | GTGGCTGACCTGGTACAAGAG |
| FADD-R | GGTAGATGCGTCTGAGTTCCAT |
| PALM-F | CGAGGTGGACGAACTCATCC |
| PALM-R | CTCCGCCGTGATGGTATCTTG |
| MYO15B-F | GCCCTGATGCGGTTTATGG |
| MYO15B-R | CTGCTTGATAACCTGGCAGTAA |
| MARC1-F | CCTCGCCTGGTCCTGATTTC |
| MARC1-R | GGCAGTAGTAGGTCCTTTGTGT |
| GPR124-F | ATCTCAGTGAATGCGAGGAACG |
| GPR124-R | GGACGACAGAGAAACATTGGG |
| LRP10-F | CCAGCCACTGATCTCCCTG |
| LRP10-R | CCAGCATAGCTGTAAGTGATGG |
| NT5C-F | ACCTGGCGGATAAAGTGGC |
| NT5C-R | GGTAGGTCGTTCATCTCCCG |
| TIMM13-F | CAGAGGATGACGGACAAGTGT |
| TIMM13-R | CATGTAGCGGTCCATGCACA |
| ENDOD1-F | GAAGCAAGCAAGCCTTGAATAC |
| ENDOD1-R | GCTAAGGGAGAATGGGTAAAGC |
| SOX12-F | AAGAGGCCGATGAACGCATT |
| SOX12-R | TAGTCCGGGTAATCCGCCAT |
| PRPF40B-F | GGATGGGATGAGTAGTGTCAACT |
| PRPF40B-R | CAAAGCTGGGGTTATCCATGAG |

Table S3. Primers for BSP

| Gene | Sequence |
| --- | --- |
| RSPO3-BSP-F | GTAGTTTTTGAGTTTAAGGGGT |
| RSPO3-BSP-R | ACAAAAAAACCAACTATTATACTTTCAAAT |
