# Supplementary material for: Upregulation of RSPO3 via targeted promoter DNA demethylation inhibits the progression of cholangiocarcinoma
Source: Clin Epigenetics. 2023 Nov 7;15:177. doi: 10.1186/s13148-023-01592-9 (PMC10629118; doi:10.1186/s13148-023-01592-9)
Supplement: Supplementary file 2 — Additional file 2. The Off-target detection of dCas9-based methylation/demethylation. [file 13148_2023_1592_MOESM2_ESM.docx]

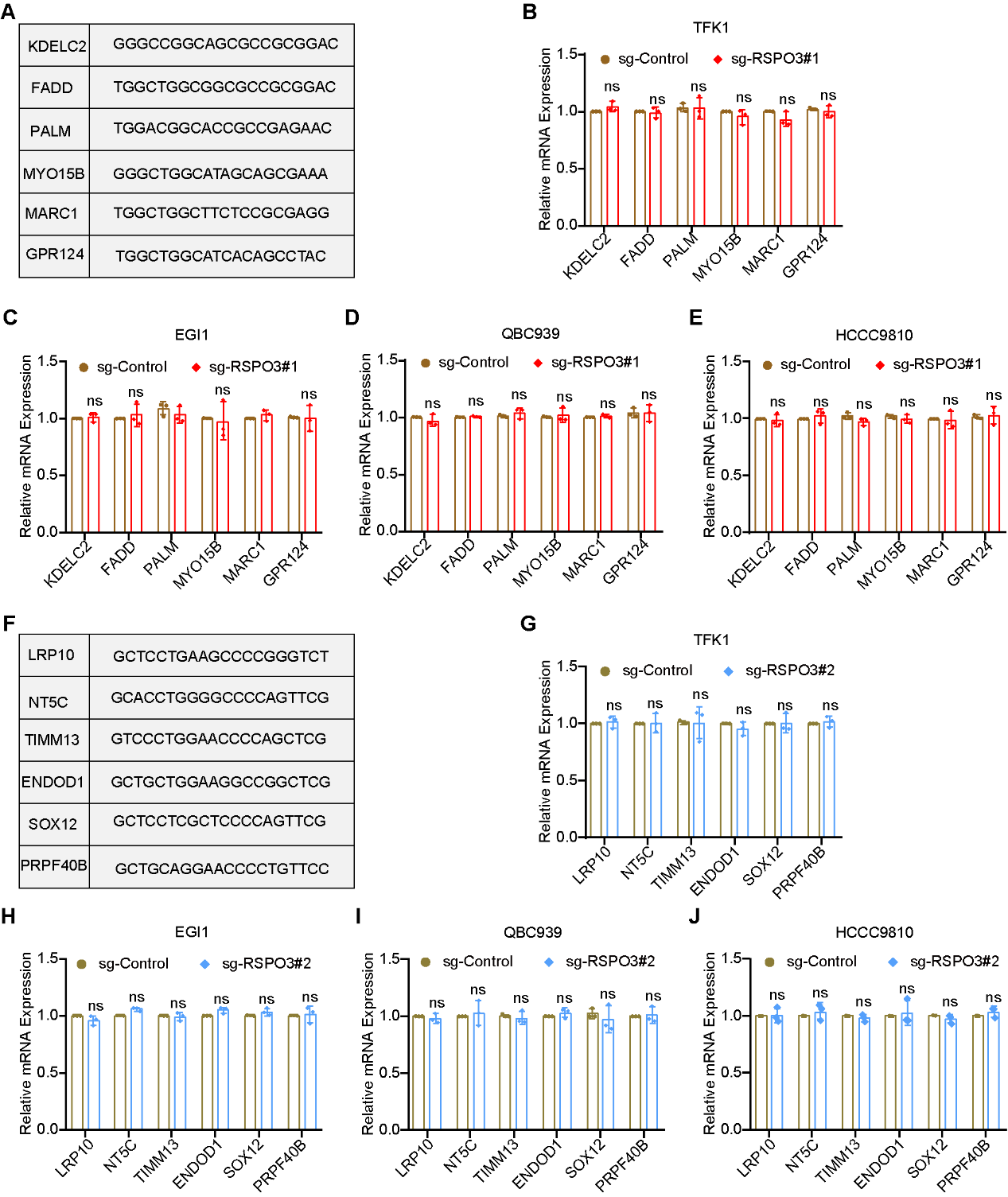

**Figure S1.** **The Off-target detection of dCas9-based methylation/demethylation.****A.F.** To evaluate the off-target effects of the dCas9-based methylation and demethylation system, we selected the top 6 potential off-target sites predicted for sgRSPO3#1 and sgRSPO3#2. **B.C.** Quantitative real-time PCR analysis of off-target mRNA expression levels in TFK1 and EGI1 cells transfected with sgRNA-dCas9DNMT3a-sgRSPO3#1. **D.E.** Quantitative real-time PCR analysis of off-target mRNA expression levels in QBC939 and HCCC9810 cells transfected with sgRNA-dCas9TET1CD-sgRSPO3#1. **G.H.** Quantitative real-time PCR analysis of off-target mRNA expression levels in TFK1 and EGI1 cells transfected with sgRNA-dCas9DNMT3a-sgRSPO3#2. **I.J.** Quantitative real-time PCR analysis of off-target mRNA expression levels in QBC939 and HCCC9810 cells transfected with sgRNA-dCas9TET1CD-sgRSPO3#2. The data was normalized to sg-control and the primer sequences can be found in Supplementary Material Table S1.
